# 27 FitBack4Literacy: Supporting Physical Literacy Journey with Physical Fitness Monitoring

**DOI:** 10.1093/eurpub/ckae114.135

**Published:** 2024-09-26

**Authors:** Gregor Jurak

**Affiliations:** University of Ljubljana, Ljubljana, Slovenia

## Abstract

FitBack, a leading European network dedicated to promoting physical fitness monitoring, will present the main result of E+ funded FitBack4Literacy project which aims to demonstrate how to properly use fitness monitoring to improve physical literacy of children and youth.

Physical literacy includes the physical competence, motivation, confidence, knowledge and understanding that people develop to maintain physical activity at an appropriate, healthy level throughout their life. Most of physical literacy interventions are focusing on physical domain, which includes physical fitness and motor skills. On the other side inappropriate fitness testing environment can result in bad experience of students and therefore also emerging stigma against fitness testing. Therefore, FitBack4Literacy project addresses both of these issues providing recommendations and educational material how to use fitness monitoring (not just testing) to build on cognitive domain of physical literacy.

Namely, the goal of FitBack4Literacy is to design and test an open, transnational digital toolkit supported by multi-language FitBack platform (www.fitbackeurope.eu) to deliver relevant information about developing physical literacy with help of fitness monitoring to teachers, coaches and adolescents.

First, teachers were involved in a participatory process to upgrade the existing FitBack reporting system by class report and learning materials. Second, consortium partners developed educational materials. Third, these materials were transformed in digital toolkit to support teachers and coaches. Finally, the upgraded FitBack reporting system, including a novel, multi-lingual digital toolkit, will be evaluated across eight European-wide sites to determine its feasibility and effectiveness in improving physical literacy with support of fitness monitoring.

Digital toolkit is technically prepared in the way to support translation of subtitles of animated topics which allows good dissemination and expend potential of its use worldwide.

